# Dietary and health biomarkers—time for an update

**DOI:** 10.1186/s12263-017-0578-y

**Published:** 2017-09-29

**Authors:** Lars O. Dragsted, Qian Gao, Giulia Praticò, Claudine Manach, David S. Wishart, Augustin Scalbert, Edith J. M. Feskens

**Affiliations:** 1 Department of Nutrition, Exercise and Sports, University of Copenhagen, Copenhagen, Denmark; 2 Department of Food Science, University of Copenhagen, Copenhagen, Denmark; 3 INRA, Human Nutrition Unit, Université Clermont Auvergne, F63000 Clermont-Ferrand, France; 4 Department of Biological Sciences, University of Alberta, Edmonton, Canada; 5 Nutrition and Metabolism Section, Biomarkers Group, International Agency for Research on Cancer (IARC), Lyon, France; 6 Division of Human Nutrition, Wageningen University & Research, Wageningen, The Netherlands

**Keywords:** Metabolomics, Biomarker, Nutrition, Ontology, Food intake, Classification, Validation, Databases, Review

## Abstract

In the dietary and health research area, biomarkers are extensively used for multiple purposes. These include biomarkers of dietary intake and nutrient status, biomarkers used to measure the biological effects of specific dietary components, and biomarkers to assess the effects of diet on health. The implementation of biomarkers in nutritional research will be important to improve measurements of dietary intake, exposure to specific dietary components, and of compliance to dietary interventions. Biomarkers could also help with improved characterization of nutritional status in study volunteers and to provide much mechanistic insight into the effects of food components and diets. Although hundreds of papers in nutrition are published annually, there is no current ontology for the area, no generally accepted classification terminology for biomarkers in nutrition and health, no systematic validation scheme for these biomarker classes, and no recent systematic review of all proposed biomarkers for food intake. While advanced databases exist for the human and food metabolomes, additional tools are needed to curate and evaluate current data on dietary and health biomarkers. The Food Biomarkers Alliance (FoodBAll) under the Joint Programming Initiative—A Healthy Diet for a Healthy Life (JPI-HDHL)—is aimed at meeting some of these challenges, identifying new dietary biomarkers, and producing new databases and review papers on biomarkers for nutritional research. This current paper outlines the needs and serves as an introduction to this thematic issue of *Genes & Nutrition* on dietary and health biomarkers.

## Background

### Introduction—biomarkers in nutrition research

Dietary and health biomarkers have been addressed in several recent reviews [1–7]. These reviews cover various applications of biomarkers in food, nutrition, and health research as well as aspects of their identification, measurement, and validation. The definition of the term “biomarker” varies considerably. While definitions in these papers cover specific aspects of food intake or health effects, biomarkers are more generally defined as “chemical or biological test results in an analysed biological material related to a certain exposure, susceptibility, or biological effect” [6]. In the Ontobee subsection on Chemical Entities of Biological Interest (ChEBI) [8], a biomarker is defined as “A substance used as an indicator of a biological state,” clearly reflecting biomarkers as a subcategory of “indicators.” “Indicators” are, in turn, defined as “anything used in a scientific experiment to indicate the presence of a substance or quality, change in a body, etc.” The ChEBI ontology therefore reflects experimental science and measurement of chemical substances as prerequisites for the use of the term, “biomarker.” However, in nutrition research, there is widespread use of observational studies and of markers that cannot be characterized as a substance, e.g., blood pressure, waist circumference, or a host antibody response. While discriminating between the terms “indicators” and “biomarkers” may be useful in some areas of research, the overlap in their definitions and use make this distinction less useful in nutrition research underlining the need for a specific ontology for nutritional science.

The distinction between different categories of biomarkers has been underlined in several reviews in the area. Jenab et al. [2] subdivides them into recovery, predictive, concentration, and replacement biomarkers, based on their biokinetics and intended use. As already mentioned, another classification divides them into exposure, effect, and susceptibility biomarkers, thereby focusing only on their use. These classifications may cause ambiguity and a unifying classification scheme may therefore be needed. This is particularly important since the discovery of new biomarkers and their validation is clearly needed to advance nutritional science as outlined in several recent reviews of this area [4, 7, 9].

Biomarker validation is particularly important in order to improve the quality of nutritional studies. However, the reliability of a biomarker may depend on the application, biological sample, sample collection strategy (time, frequency), and study design. A clear distinction of validation criteria for the different classes of biomarkers is therefore needed.

**Fig. 1:**
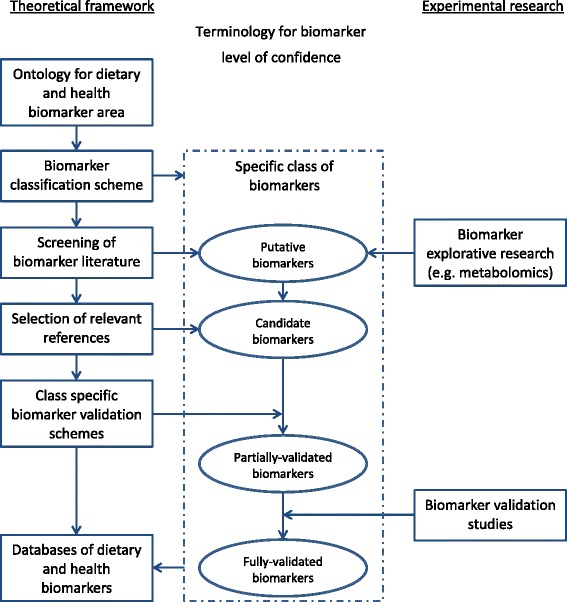
A schematic overview of a framework supporting the development of dietary biomarkers. Ontology and classification scheme serve as the tools to navigate the targeted class of biomarkers. For each specific class of biomarkers, literature search would be conducted to provide reviews of the current state of knowledge on putative biomarkers. Putative biomarkers may also be identified by new explorative research. Candidate biomarkers are selected from the putative biomarkers by removing implausible entries based on literature. A validation scheme is applied on the candidate biomarkers to assess their validity by a defined set of criteria to identify the most promising candidate biomarkers as partially or fully validated for a specified use. Further validation studies may be used to systematically validate the best candidate biomarkers. All the available information is shared in public databases to support further studies on the development of biomarkers

Excellent tools and guidance exist for producing systematic reviews and meta-analyses such as the Cochrane handbook [10]. In addition, the PRISMA Statement [11, 12] has been developed to assist researchers in conducting systematic reviews of randomized trials and interventions. When it comes to biomarkers used as tools for measuring food intake or assessing nutritional status, there is a need for another paradigm because several of the steps described for the current procedures do not apply. Also, when it comes to sharing all of this information in databases and associated online tools, there is a need to build upon several of the tools already mentioned. These include ontologies for the subject area, a classification scheme for biomarkers, validation tools, and high quality reviews of the current state of knowledge, see Fig. 1. As a project launched under the JPI-HDHL, the FoodBAll consortium aims to close some of these gaps through a series of reviews in this thematic issue of the journal.

### An ontology for the dietary and health biomarker area

**Fig. 2:**
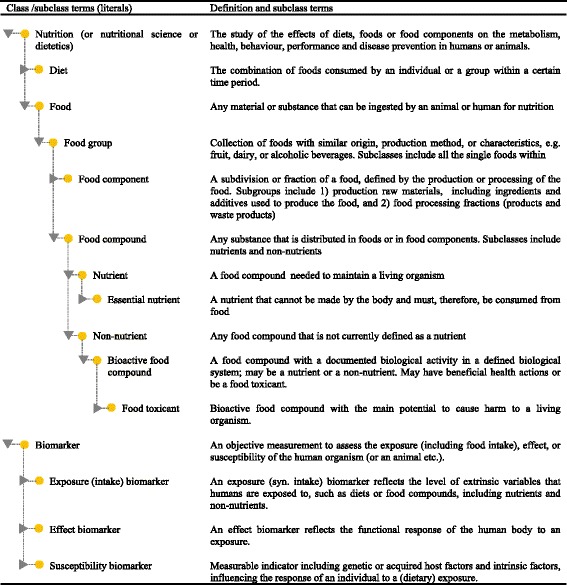
Proposed terms for initiating ontology for the dietary and health biomarker area

Ontologies exist for several nutrition-related areas, including biological chemistry [8] and environmental [8], bioassay [13], and biomedical investigations [14]. There is even some initial work on an ontology for nutritional studies [15] and an ontology for food [16]. However, most terms and relationships related to nutrition and biomarkers are not yet covered at any of these sites. Creating a network of defined terms with connections to some of these ontologies is therefore a potential way forward. It is not the intention here to formally develop full nutrition ontology, only to define terms that can serve as classes and subclasses in developing ontology for this field. Figure 2 contains suggestions for terms that could be included in such nutrition ontology and at the same time outlines the definitions of terms used in this thematic issue of *Genes and Nutrition*.

The connection of the term “food” to the simple definition in ChEBI (“Any material that can be ingested by an organism”) is useful. However, it is a bit too broad by not excluding ingestion of drugs or non-food objects. It is further complicated because this ontology has organized the term as a subclass of “food component,” while it would be more useful to have food as class term, with food components and food compounds as subclasses as we suggest here. In ENVO (the Environment Ontology), the term “food product” is defined as “A substance, usually composed primarily of carbohydrates, fats, water and/or proteins, that can be eaten or drunk by an animal or human being for nutrition or pleasure” [8]. Defining food products solely as substances may be confusing in chemistry-related fields such as nutrition and food chemistry, so using the term “material” to define food should be preferred. Since many non-foods such as drugs could be ingested “for pleasure,” the definition is also a bit too broad and the complex practices, frequency, or reasons for ingesting foods should be avoided in the definition. Therefore, in simplicity, any material may be considered a food as long as it is inherently able to sustain nutrition to some extent. The preferred definition of food for nutrition and health science would therefore be “Any material or substance that can be ingested by an animal or human for nutrition.” The subclasses of “food products” in ENVO should also be subclasses of “food” and “food group” thereby linking downstream to the various single foods and food components for which biomarkers of intake should ideally be found.

Nutrition, as such, is not found as a term in any ontology yet while “nutritional science” is an undefined class term with no subclasses under biomedical science in EMBRACE [17]. A broad definition of nutritional science suggested here is “The science of all processes by which organisms take in and utilise nutrients or other food components,” while nutrients could be defined as “Food compounds needed to maintain a living organism.” Note that nutritional science as defined here also embraces non-nutrient components in the food since these compounds may have considerable influence on the health effects of foods. This is also the case for public definitions such as the one found in Wikipedia, “Nutrition is the science that interprets the interaction of nutrients and other substances in food in relation to maintenance, growth, reproduction, health and disease of an organism” [18]. Food intake and nutrition are closely related therefore making “diet” a natural link between the food science terms and the nutrition area. In this case, “diet” may therefore in this context be defined as “The combination of foods consumed by an individual or a group within a certain time period.” By defining these terms and linking them with existing ontologies, dietary and health biomarkers can now be discussed on the basis of a coherent set of terms.

### Ambiguity of biomarker classifications

There are a number of biomarkers that may belong to several of the classes described by Biesalski et al. [6], i.e., they can be used as exposure, effect, or susceptibility biomarkers, depending on the study purpose and design. Several of the exposure biomarkers measured in plasma, which are termed concentration and replacement biomarkers by Jenab et al. [2], may be used to assess exposures to nutrients or contaminants. However, for some of these compounds such as vitamins, minerals, or heavy metals, there are also established thresholds for minimal or maximal concentrations beyond which there is an increased risk of deficiency (for vitamins and minerals) or toxicity. When biomarkers are used to compare sample concentrations with such limits, they are actually used to assess the status, vulnerability, or even risk of an individual and, hence, should be classified as susceptibility biomarkers. Clearly, the classification in these cases is more dependent on the intended application of the measurements than on the methodology as such. Exposure biomarkers in urine have been termed recovery biomarkers if the full dose may be recovered. Alternately, they are called predictive biomarkers if only a fraction is excreted [2]. If a dietary treatment is used to improve absorption of a nutrient, then this marker becomes related to response rather than exposure or susceptibility. Most metabolites measured in urine may therefore qualify in each of the major classes, depending on the purpose of the measurements and study design. For example, p-cresol sulfate along with other metabolites would result from environmental exposure to p-cresol; however, this compound is also formed endogenously by our microbiota. Formation is clearly affected by the composition of the diet in terms of omnivorous and vegetarian diets and hence may be said to reflect dietary intake [19]. On the other hand, p-cresol formation may also affect sulfation capacity so its sulfate ester may be a marker of altered metabolism (effect) or residual capacity (susceptibility) [20]. Additionally, p-cresol sulfate has been shown to be a susceptibility marker related to risk of progressing kidney disease [21]. In other words, p-cresol sulfate as a biomarker could have at least three different classifications, depending on the intended use. Many other exposure biomarkers, including omega-3 fatty acids, beta-carotene, and choline metabolites also reflect some degree of functional change or host factor capacity leading to similar biomarker classification ambiguity. Other examples, some of which will be discussed below, include measurements of blood pressure, blood glucose, and hippuric acid. Biomarker ambiguity, whether biochemical, anthropometric, or physiological, is therefore quite common, as many combine elements of two or three of the exposure, effect, and susceptibility marker classes. Biomarkers are typically affected by a combination of exposures and host factors and consequently complex to interpret, resulting potentially in controversy. Blood pressure may serve as an illustrative example. It is well established today that blood pressure is influenced by genetic (host) factors and genetic variation may be involved in 50 % of the population variability [22]. Blood pressure is also affected by dietary and lifestyle exposures, including exercise [23], smoking [24], and healthy eating [25]. While relationships with risk of stroke and coronary disease is quite clear, health-related effects of blood pressure within the normal range from 120/80 to − 90/60 mmHg are not equally clear and a large variation in what constitutes an optimal blood pressure may exist on an inter-individual basis [26]. Moreover, the measurement is very sensitive to the protocol and repeated measurements should be done by the same person. Care must therefore be exercised in study planning and in interpretation when blood pressure is used as a marker, and it should be clear whether it is used for determining risk or effect. In analogy to blood pressure, there is a range of biochemical and physiological biomarkers where only the high and low ends of the outcome scale are readily interpreted in terms of individual risk, e.g., most anthropometric measures, hormones, micronutrients, intermediary metabolites, and cognitive scales.

An important consequence of this ambiguity is that validation of a biomarker may depend on its use. Most validation schemes can roughly be subdivided into analytical performance and biological interpretability. The analytical performance of a biomarker may often be independent of the study design and purpose. However, this is clearly not the case when the measurement of extremes is more important than the normal range for biological reasons. For instance, the detection limit or linear range of a method may suffice for an assessment of baseline characteristics but not for the assessment of an extreme response or vice versa. For instance, the use of glucose monitors may reflect variation with sufficient precision to follow the change in response in individuals during an OGTT or dietary test (i.e., used as an effect biomarker), while the accuracy of the same method would not suffice to determine fasting glucose levels for diagnostic purposes (i.e., as a risk or susceptibility biomarker). Validation of biomarker measurements may therefore depend on the biomarker class, which in turn may depend mainly on its intended use. Validation schemes taking into consideration the intended applications of dietary and health biomarkers are therefore needed in order to help validate the large number of new potential biomarkers resulting from the many explorative (“omics”) investigations on diet and health.

### Analysis of the literature for assessing biomarker validity

Putative new biomarkers of dietary exposures and of dietary effects on health are being published at a rapid pace as a result of recent developments in metabolomics [4, 27], but previous work through the last 40+ years has also pointed to a number of potentially important dietary biomarker compounds identified by more traditional approaches. Some of these have been “re-discovered” by metabolomics. This calls for standards for doing systematic literature searches and for evaluating biomarker candidates. Standards for systematic reviews and meta-analyses already exist for effect markers, including the Cochrane guidelines [10]. The PRISMA statement [11] also helps to assess many aspects of individual study quality in order to weigh their importance for an overall conclusion. These aspects relate to the strength of the experimental or observational designs, the quality of recruitment, the randomization procedures, etc. The aim of these guidelines are to critically assess effects reported in human studies and they are not aimed at assessing methodological studies or performing systematic reviews for food and dietary intake biomarkers. Guidelines developed specifically for assessing the literature on biomarkers are therefore needed. The aim should be to find previously suggested biomarkers and to critically assess their quality. Moreover, the evaluation of each biomarker candidate should be supported by the literature search strategy by including different quality aspects. The vision for this work on intake biomarkers would be:

- to identify and evaluate existing putative intake biomarkers for all food groups based on the literature,
- to validate the more promising candidates using a coherent quality assessment scheme, and
- to create a database including all suggestive food intake biomarkers along with their current level of validity for assessing exposure.

This should support further work on food intake biomarker development and validation by pointing out the studies needed to improve the assessment of validity. Moreover, such a system should help researchers to assess the quality of food intake biomarkers that are considered for use in human studies on diet and health. Similar literature search guidelines, quality assessment tools, and validation schemes need also to be developed for susceptibility and effect biomarkers.

### Supporting databases for food intake biomarkers

Biomarker development for research in nutrition and health is dependent on resources to quickly find information on compounds in foods and on food intake biomarkers proposed by others. The literature review and validation of biomarkers for all major food groups should therefore be entered into searchable database structures along with unique identifiers. The most comprehensive databases on food constituents and their chemical and biological data are FooDB (www.foodb.ca) [28], the expert-curated database PhytoHub (www.phytohub.eu) [29] focused on dietary phytochemicals, and the Phenol-Explorer database on polyphenols [30]. These databases are currently being enriched to include new data on food non-nutrients and their human metabolites. The added metabolites will include the known metabolites described in the literature as well as in silico predicted metabolites, thereby covering large numbers of potential biomarkers for food intake. In parallel, a new database called Exposome-Explorer (exposome-explorer.iarc.fr) is being developed to include all known dietary biomarkers and rich information on their measurement in various populations [31]. Exposome-Explorer will thereby supplement information in the human metabolome database [32].

Adding mass spectral and other information is of central importance to help researchers annotate findings from metabolite profiling studies. In many cases, the compounds measured as biomarkers are not commercially available and information on their (bio)synthesis and availability in non-commercial laboratories for sharing can be found in FoodComEx (Food Compound Exchange, foodcomex.org) [33]. FoodComEx is designed as an online catalog of pure compounds, which have been made available by academic laboratories. Exchange of compounds with a provider depends on bilateral agreements on the terms of collaboration. Rules for these collaborations have been defined in a charter of good practices. FoodComEx is a collaborative initiative widely open to new contributors and users.

Another web resource developed in the FoodBAll project is a web portal (foodmetabolome.org) [34]. This portal is continuously updated to present links to the most useful tools, databases, libraries of spectra, and software for nutritional metabolomics as well as for dietary biomarker discovery. The portal will be further developed to present tutorials, webinars, and news related to the food metabolome and to food intake biomarkers.

## Conclusions

The current pace of biomarker discovery and biomarker applications is higher than ever before due to the rapid development of “omics” technologies and data collection. This rapid development may reshape future research in nutrition and health. In order to support this development, there is a need to develop ontologies for food, nutrition, and diet-related health areas. There is also a need to classify biomarkers in such a way that systematic attempts to validate them and develop them into trusted research tools is possible according to standardized criteria and according to their intended use. Finally, there is a need for improved methods to systematically search both older and more recent literature for the best biomarkers for foods, food groups, and food constituents and to develop and support database systems to include updated information on the validity of biomarker measurements for different applications. All of these aspects are addressed in this special issue of *Genes and Nutrition* by partners of the FoodBAll consortium.
